# Dietary Milk Sphingomyelin Prevents Disruption of Skin Barrier Function in Hairless Mice after UV-B Irradiation

**DOI:** 10.1371/journal.pone.0136377

**Published:** 2015-08-24

**Authors:** Chisato Oba, Masashi Morifuji, Satomi Ichikawa, Kyoko Ito, Keiko Kawahata, Taketo Yamaji, Yukio Asami, Hiroyuki Itou, Tatsuya Sugawara

**Affiliations:** 1 Food Science Research Labs, Meiji Co., Ltd., 540 Naruda, Odawara-shi, Kanagawa, 250–0862, Japan; 2 Division of Applied Biosciences, Graduate School of Agriculture, Kyoto University, Kitashirakawaoiwakecho, Sakyo-ku, Kyoto, Kyoto, 606–8502, Japan; University Hospital Hamburg-Eppendorf, GERMANY

## Abstract

Exposure to ultraviolet-B (UV-B) irradiation causes skin barrier defects. Based on earlier findings that milk phospholipids containing high amounts of sphingomyelin (SM) improved the water content of the stratum corneum (SC) in normal mice, here we investigated the effects of dietary milk SM on skin barrier defects induced by a single dose of UV-B irradiation in hairless mice. Nine week old hairless mice were orally administrated SM (146 mg/kg BW/day) for a total of ten days. After seven days of SM administration, the dorsal skin was exposed to a single dose of UV-B (20 mJ/cm^2^). Administration of SM significantly suppressed an increase in transepidermal water loss and a decrease in SC water content induced by UV-B irradiation. SM supplementation significantly maintained covalently-bound ω-hydroxy ceramide levels and down-regulated mRNA levels of acute inflammation-associated genes, including thymic stromal lymphopoietin, interleukin-1 beta, and interleukin-6. Furthermore, significantly higher levels of loricrin and transglutaminase-3 mRNA were observed in the SM group. Our study shows for the first time that dietary SM modulates epidermal structures, and can help prevent disruption of skin barrier function after UV-B irradiation.

## Introduction

Skin provides an effective barrier between the organism and the environment by helping to reduce the risk of physical, chemical, and microbial damage. Exposure to ultraviolet-B (UV-B) radiation is a key factor in the initiation of photo-aging, which can be characterized by dryness, wrinkling, and mottled pigmentation [1–3]. UV irradiation of mammalian skin disrupts epidermal permeability barrier functions, and is accompanied by an increase in transepidermal water loss (TEWL) [4–6] as well as alterations in the stratum corneum (SC) lipid profile [7, 8].

Reduced barrier function appears to be a consequence of inadequate structural conditions in the epidermis. Skin barrier properties are primarily localized in the SC, which is the outermost layer of the epidermis. The SC consists of corneocytes surrounded by an intracellular matrix that is enriched in neutral lipids. Ceramides, which comprise approximately 50 mass-% of intercellular lipids, play an important role in retaining epidermal water and, in combination with cholesterol and free fatty acids, influence the permeability of the epidermal barrier [7, 9]. The SC also contains covalently-bound ω-hydroxy ceramides. These ceramides are most frequently bound via ester linkages to structural proteins in the epidermal cornified envelope (CE), which is a critical permeability barrier structure in the SC [10–13]. Previous studies showed that levels of covalently-bound ceramides, but not unbound-ceramides, were significantly reduced in parallel with a marked increase in TEWL following irradiation with a single UV-B dose in hairless rodents [7, 8]. Furthermore, the CE is formed during terminal differentiation of the epidermis through crosslinking of specific precursor proteins, including involucrin, loricrin, small proline-rich proteins, and transglutaminase (TGase), which are essential for skin barrier function [13, 14]. Therefore, lipid species such as ω-hydroxy ceramides, together with CE components are thought to play a crucial role in the formation of lamella structures, and are involved in maintaining skin barrier functions. However, few studies have been able to demonstrate that the oral intake of dietary components can modulate epidermal structures associated with dryness induced by UV-B irradiation.

Dietary components are known to play beneficial roles in improving disrupted skin barrier functions [15–17]. In bovine milk, phospholipids represent approximately 0.5% to 1% of the total lipid content, and mainly consist of sphingomyelin (SM) and phosphatidylcholine. Phospholipid concentrates containing SM prepared from bovine milk have been shown to increase SC hydration and reduce TEWL in hairless mice fed a standard diet [18, 19]. Haruta-Ono et al. reported that orally administrated sphingomyelin incorporated into skin sphingomyelin and converted to stratum corneum ceramide [20]. *In vivo* study showed that sphingoid base, sphingosine, improves transepithelial electric resistance value in SDS treated-keratinocytes [21]. However, the mechanisms by which milk SM improve skin barrier functions remain unclear. Therefore, in present study we investigated the effects of dietary milk SM on skin barrier defects induced by a single dose of UV-B irradiation in hairless mice.

## Materials and Methods

### Animals

Sixty four nine-week-old female hairless mice (Hos: HR-1, Nippon SLC Inc., Shizuoka, Japan) were used in this study. All mice were housed in plastic cages (four mice/cage) in a temperature- and humidity-controlled room (24 ± 1°C and 50 ± 10% relative humidity [RH]) under a 12 hr light-dark cycle. Mice were allowed free access to the standard diet AIN-93G (Oriental Yeast Co., Ltd., Tokyo, Japan) and water. All of the animal experiments in this study were approved by Meiji Co., Ltd. Institutional Animal Care and Use Committee, and performed in accordance with the Guiding Principles for the Care and Use of Laboratory Animals approved by Meiji Co., Ltd (Permit Number: 2013_3871_0082). All surgery was performed under isoflurane anesthesia, and all efforts were made to minimize suffering.

### Experimental design

After acclimatization for four days, mice were randomized into eight groups (control groups [day 0, 1, 2, 3] and SM groups [day 0, 1, 2, 3]), according to body weight, TEWL, and SC water content. The control group was given 5% ethanol solution at 10 mL/kg body weight, while the other groups were given SM at 146 mg/5% ethanol solution at 10 mL/kg body weight. SM from milk (certified ≥ 98% purity, NS220204) was purchased from Nagara Science Co., Ltd. (Gifu, Japan).

Mice were given the experimental SM samples orally for 10 days, from 1 week before UV-B irradiation (day -7) until 3 days after irradiation (day 3). One week after the initiation of the experimental sample administration (day 0), the dorsal skin was exposed once to 20 mJ/cm^2^ emitted by a UV-B lamp (GL20SE, Sankyo Denki Co., Ltd., Tokyo, Japan) under isoflurane anesthesia. TEWL and SC water content were measured 7 days before and 0, 1, 2, and 3 days after irradiation [17]. All mice were euthanized under isoflurane anesthesia on days 0, 1, 2, or 3. The dorsal skin was excised quickly and immediately frozen at -80°C until analysis.

### Measurement of TEWL and SC water content

TEWL and SC water content were assessed under standardized conditions (external temperature 24 ± 1°C and 50 ± 10% RH) using a Tewameter MPA580 (Courage and Khazaka Electronic GmbH, Cologne, Germany) and SKICON 200-EX (I.B.S Co., Shizuoka, Japan) apparatus, respectively.

### Total RNA isolation, cDNA synthesis and quantitative real-time reverse transcription polymerase chain reaction (RT–PCR) analysis

Dorsal skin samples from each mouse were frozen in liquid nitrogen and powdered. Total RNA was isolated from the skin samples using the guanidine thiocyanate method [22] with TRIzol reagent (Life Technologies Corporation, Carlsbad, CA, USA), and purified with an RNeasy Mini Kit (Qiagen, Hilden, Germany). Extracted RNA was then dissolved in diethylpyrocarbonate-treated water and quantified spectrophotometrically at a wavelength of 260 nm. Reverse transcription was performed using a RivertAid First Strand cDNA Synthesis Kit (Thermo Fisher Scientific, Waltham, MA, USA). The cDNA was stored at -80°C prior to subsequent analysis.

Quantitative Real-time PCR was performed using the ABI 7500 Fast Realtime PCR system (Applied Biosystems, Foster City, CA, USA). The respective primers and probes (TaqMan Gene Expression Assays) were designed at Applied Biosystems from gene sequences obtained from GenBank (thymic stromal lymphopoietin [TSLP]: Mm01157588_m1, IL-1 beta: Mm00434228_m1, IL-6: Mm00498375_m1, loricrin: Mm01962650_s1, TGase-3: Mm00436999_m1, glyceraldehyde-3-phosphate dehydrogenase [GAPDH]: Mm99999915_g1). The relative expression of the gene of interest was normalized relative to GAPDH mRNA levels and then calculated using the 2^-ΔΔ^Ct method [23]. The results are expressed as arbitrary units.

### Extraction of covalently-bound ceramides

Extraction of covalently-bound ceramides followed a modified version of the method reported by Macheleidt *et al*. [24]. Epidermal sheets were obtained from the skin samples by overnight incubation with Dispase II (Roche, Indianapolis, IN, USA) at 4°C. Tissues were homogenized in chloroform/methanol (2:1, v/v) using a glass homogenizer. After removal of the supernatant by centrifugation, the protein residue was washed twice with a chloroform/methanol solution until unbound ceramides were not detectable. After drying, the protein pellets were incubated overnight in 1 M KOH in 95% methanol at room temperature to release the lipids that were covalently-bound to the SC by ester-like bonds. The methanolic layer was then removed after centrifugation and neutralized with 1 N HCl. The resulting protein pellets were washed using chloroform/methanol (2:1, v/v). The organic phases were combined, dried, and redissolved in methanol while the protein pellet was resuspended in 0.1 M sodium hydroxide containing 1% sodium dodecyl sulfate solution and incubated at 60°C for 2 hr to solubilize the proteins. After incubation, the solution was neutralized with 1 N hydrochloric acid. Protein concentrations were determined using a commercial kit (Micro BCA assay kit, Pierce Biotechnology, Inc., Rockford, IL, USA).

### Analysis of covalently-bound ceramide content by HPLC/MS/MS

Covalently-bound ceramides in mouse epidermal tissues were identified using a system for high performance liquid chromatography coupled to a tandem mass spectrometer (HPLC/MS/MS) (Quattro Premier XE, Waters Corporation, Milford, MA, USA) [25]. All analyses were performed on a 2 X 100 mm column with a particle size of 1.7 μm (ACQUITY UPLC BEH C18, Waters Corporation). The mobile phase A consisted of 5 mM ammonium acetate in 95% methanol, while the mobile phase B consisted of 5 mM ammonium acetate in acetonitrile. The initial eluent composition was 100% A, followed by an increase to 100% B over 30 min, 100% B for 2 min, and then a reduction to 0% A over 3 min. The total running time was 35 min with an eluent flow of 0.4 mL/min and a column temperature of 40°C. Analytes were detected using electrospray ionization in the positive mode. Multiple-reaction-monitoring (MRM) was performed using characteristic fragmentation ions (*m/z* 804.8/264.3 for major covalently-bound ω-hydroxy ceramide, ω-hydroxy tetratriacontenoic-sphingosine [d18:1- ω-hydroxy C34:1]). The parameters for the HPLC/MS/MS analysis of were as follows: capillary voltage, 3000 V; source temperature, 120°C; desolvation temperature, 400°C; desolvation gas flow, 850 L/hr; cone gas flow, 50 L/hr; cone voltage, 40 V and collision energy, 30 eV. From the epidermis of hairless mice, we identified eleven molecular species of protein-bound ω-hydroxy ceramides which consisted of d18:1-C30:0, d18:1-C32:0, d18:1-C32:1, d18:1-C34:0, d18:1-C34:1, d18:1-C36:1, d17:1-C32:0, d17:1-C32:1, d17:1-C34:0, d17:1-C34:1, and d17:1-C36:1. Molecular species of ceramide, ω-hydroxy tetratriacontenoic-sphingosine (d18:1-C34:1), was the main component in the epidermis [25]. Data are expressed as the relative peak area per epidermal protein content.

### Statistical analysis

All data are presented as means ± standard deviation (SD). The real-time PCR results were expressed as fold change ± SD. Data were analyzed by two-way ANOVA with post hoc analyses being carried out using Dunnett’s test (time) and Student’s t-test (group) (SPSS ver. 22.0, SPSS, IL, USA). The statistical analyses of gene expression were performed at the ^Δ^Ct stage in order to exclude potential bias due to the averaging of data transformed through the equation 2^-ΔΔCt^ [26]. Differences among groups were considered to be significant at *P* < 0.05.

## Results

### Effect of dietary SM on TEWL and SC water content induced by a single dose of UV-B irradiation

**Fig 1:**
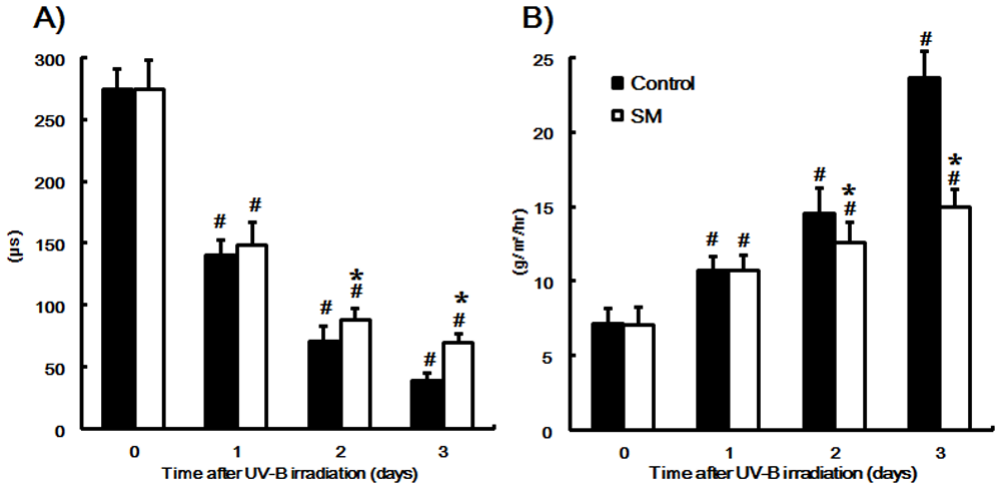
Effect of sphingomyelin on TEWL and SC water content (B) after UV-B irradiation. (A) Transepidermal water loss (TEWL); (B) Stratum corneum (SC) water content. The values are shown as mean ± SD (n = 8/group). *: *P* < 0.05 (vs. the control group). #: *P* < 0.05 (vs. day 0).

TEWL and the water content of SC are shown in Fig 1. A single dose of UV-B irradiation significantly increased TEWL (Fig 1A) and decreased the SC water content (Fig 1B) at every time point assessed. TEWL was significantly lowered in the SM group relative to the control group on days 2 and 3 after irradiation. Meanwhile, the water content of the SC was significantly higher in the SM group than the control group on days 2 and 3.

### Effect of dietary SM on covalently-bound ω-hydroxy ceramide levels

**Fig 2:**
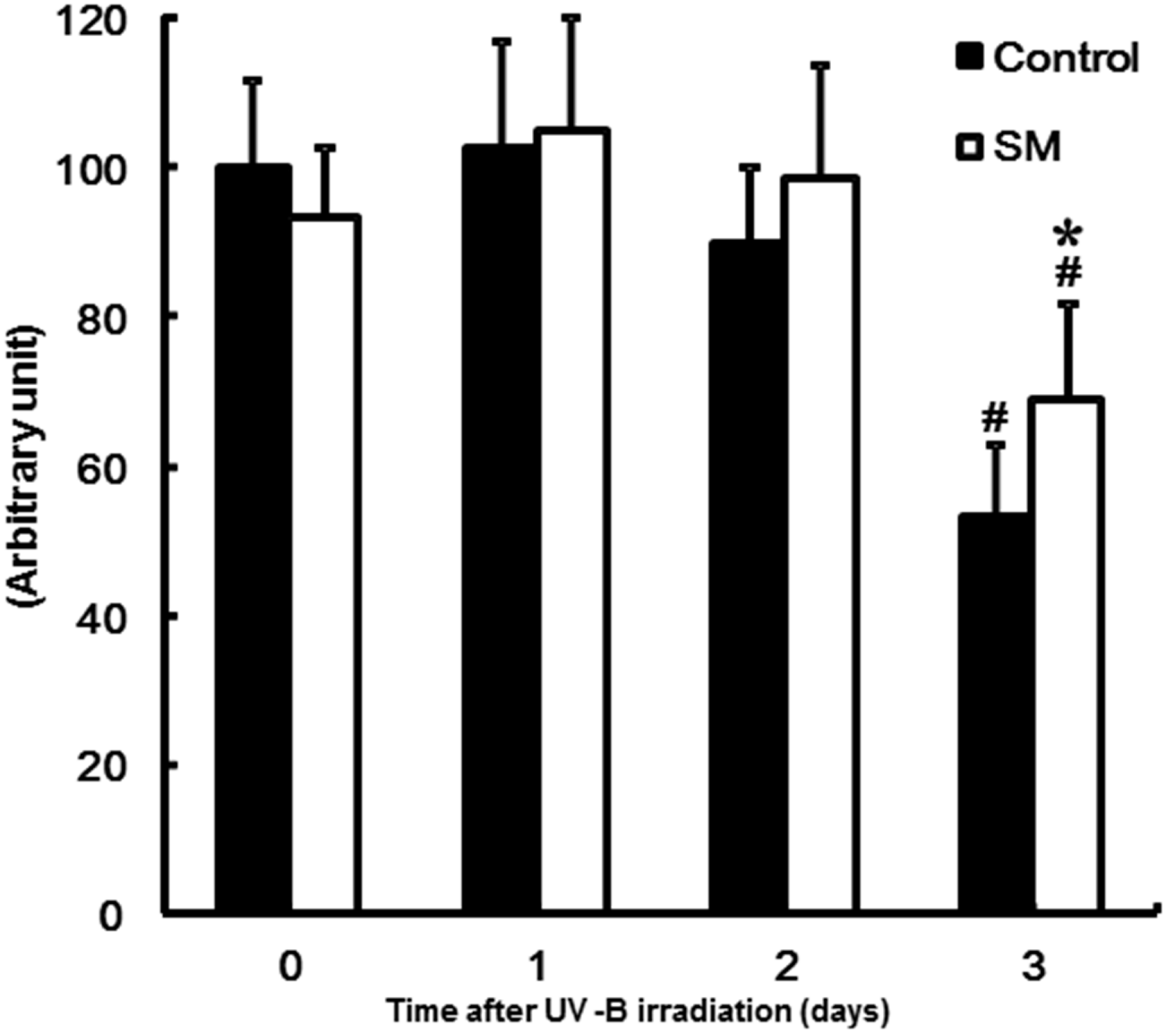
Effect of sphingomyelin on levels of covalently-bound ω-hydroxy ceramide in the epidermis induced by UV-B irradiation. Levels of ω-hydroxy tetratriacontenoic sphingoshine (d18:1-ω-hydroxy C34:1) are expressed as relative peak area per epidermal protein content. The values are shown as mean ± SD (n = 8/group). *: *P* < 0.05 (vs. the control group). #: *P* < 0.05 (vs. day 0).

Levels of ω-hydroxy tetratriacontenoic sphingoshine (d18:1-ω-hydroxy C34:1) in the epidermis are shown in Fig 2. On day 3 after UV-B irradiation, the level of covalently-bound ω-hydroxy ceramide was significantly decreased in both groups. However, the amount of covalently-bound ω-hydroxy ceramide was significantly lower in the control group relative to the SM group.

### Effect of dietary SM on mRNA levels of skin inflammation-related genes

**Fig 3:**
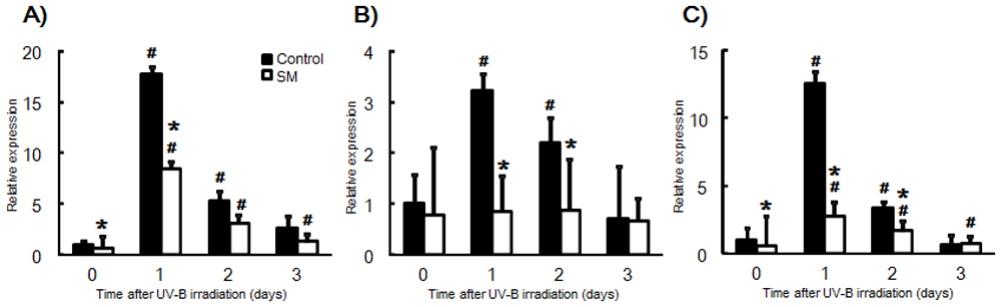
Effect of sphingomyelin on expression levels of TSLP (A), IL-1 beta (B) and IL-6 (C). (A) Thymic stromal lymphopoietin (TSLP); (B) Interleukin-1 (IL-1) beta; (C) Interleukin-6 (IL-6). The mRNA levels are expressed as fold-change ± SD (SD of ^Δ^Ct) (n = 8/group). *: *P* < 0.05 (vs. the control group). #: *P* < 0.05 (vs. day 0).

A significant up-regulation of TSLP, IL-1 beta, and IL-6 mRNA was observed in the control group on day 1 and 2, while TSLP and IL-6 mRNA levels were significantly increased in the SM group at every time point assessed (Fig 3). There was no change in the levels of IL-1 beta mRNA in the SM group after UV-B irradiation. The mRNA level of TSLP was significantly higher in the control group than the SM group on days 0 and 1, while the mRNA level of IL-1 beta on days 1 and 2 was markedly decreased for the control group and relatively constant for the SM group (Fig 3A and 3B). Meanwhile, IL-6 levels increased in the control group on day 1 after UV-B irradiation before tapering on days 2 and 3, while the SM group showed a similar expression pattern but with lower levels of mRNA (Fig 3C).

### Effect of dietary SM on mRNA levels of epidermal structure-related genes

**Fig 4:**
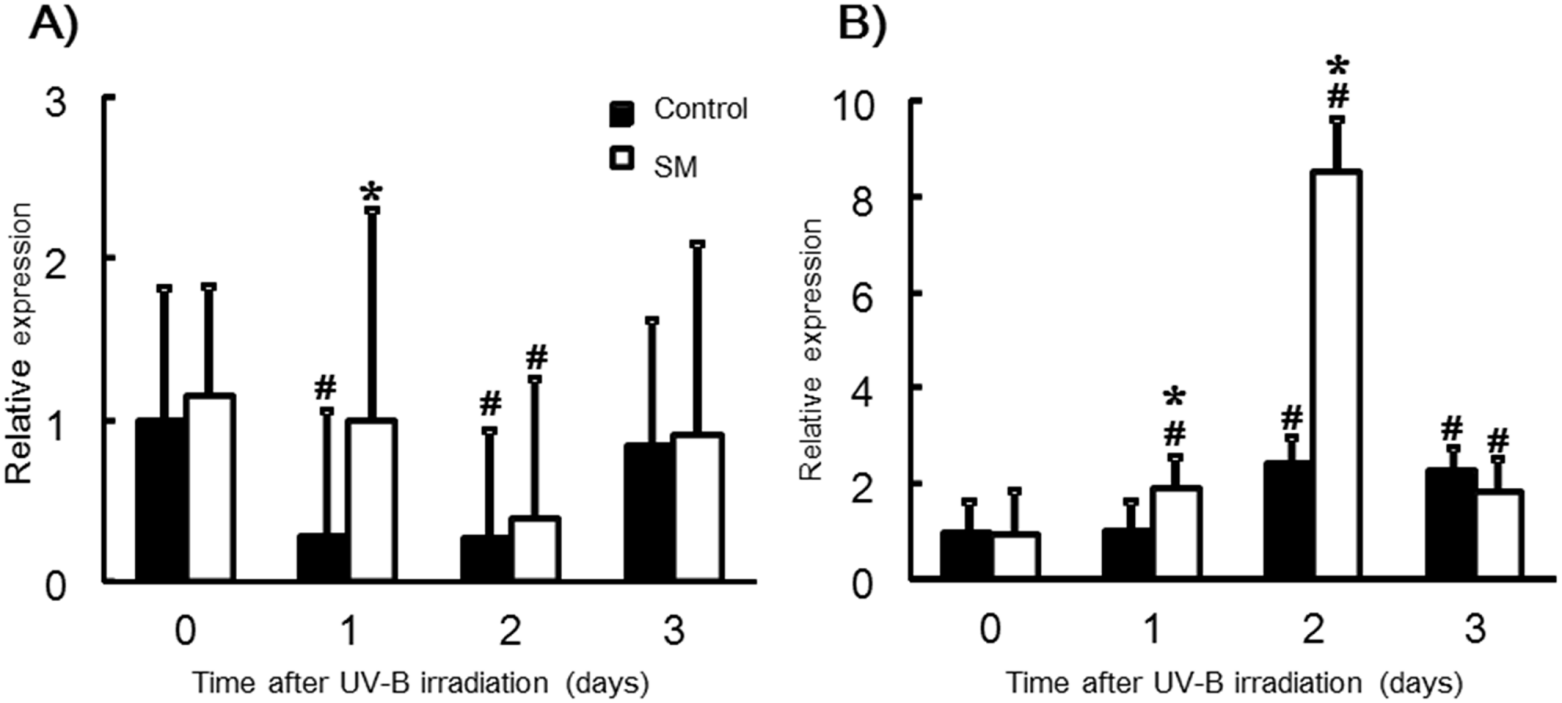
Effect of sphingomyelin on Loricrin (A) and TGase-3 (B) mRNA expression. (A) Loricrin; (B) Transglutaminase-3 (TGase-3). The mRNA levels are expressed as fold-change ± SD (SD of ^Δ^Ct) (n = 8/group). *: *P* < 0.05 (vs. the control group). #: *P* < 0.05 (vs. day 0).

Loricrin and TGase-3 are known as enhancement factors of CE structures in the epidermis. Levels of loricrin mRNA exhibited a significant decrease in the control group on days 1 and 2 after UV-B irradiation, while for the SM group loricrin mRNA levels were maintained until day 2 (Fig 4A). The mRNA level of loricrin was significantly increased in the SM group than in the control group on day 1 after UV-B irradiation. The mRNA level of TGase-3 showed a significant increase in the control group on days 2 and 3, while for the SM group increased levels were observed on day 1 and particularly day 2 (Fig 4B). The mRNA level of TGase-3 showed a significant higher in the SM group than in the control group on days 1 and 2.

## Discussion

A single UV-B irradiation dose has long been known to cause skin barrier dysfunction as well as increased TEWL and decreases in the water content of the SC. Here we demonstrated for the first time that dietary SM could prevent the disruption of skin barrier function in hairless mice after a single dose of UV-B irradiation. Furthermore, SM administration suppressed a decrease in both the amount of protein-bound ω-hydroxy ceramides and loricrin mRNA levels. Together, these results suggest that changes in epidermal structure markers such as covalently-bound ω-hydroxy ceramide content and CE formations seen in in mice fed milk SM might be associated with an improvement in skin barrier defects that are induced by UV-B irradiation.

One possible explanation for this attenuation of skin barrier defects is that dietary SM relieves skin inflammation, thereby promoting a decrease in the levels of covalently-bound ω-hydroxy ceramide. UV-B irradiation is known to cause a decrease in ω-hydroxy ceramide levels in parallel with a significant increase in TEWL, however, the content of unbound ceramide increases [8]. Therefore, covalently-bound ω-hydroxy ceramides in the epidermis are thought to play an important role in the formation of lamellar structures that participate in epidermal water retention. We characterized the covalently-bound ω-hydroxy ceramides from murine epidermises using HPLC-MS/MS and identified eleven species of covalently-bound long-chain (C30-36) ω-hydroxy ceramides containing either a C18:1 or C17:1 sphingoid base (data not shown). The major molecular species of covalently-bound long-chain ω-hydroxy ceramides was ω-hydroxy tetratriacontenoic-sphingosine (d18:1-ω-hydroxy C34:1), and the levels of this ceramide were significantly decreased after UV-B exposure.

UV-B exposure facilitates release of pro-inflammatory mediators from various skin cells as well as the activation and subsequent infiltration of immune cells into the skin. The UVB-induced immune response usually begins with an abnormal release of inflammatory cytokines, including IL-1, IL-6, IL-8, and tumor necrosis factor-α (TNF-α) in keratinocytes [27–30]. The results of this study are in agreement with these previous findings in that mRNA levels of the inflammation-associated genes TSLP, IL-1β and IL-6 were acutely up-regulated one day after a single dose of UV-B irradiation prior to the manifestation of epidermal barrier dysfunction. Recent research showed that in human keratinocytes Th-2 cytokines such as IL-4 and IL-6 markedly decreased the production of esterified ω-hydroxy ceramides, which are precursors of covalently bound ω-hydroxy ceramides [31]. Therefore, skin inflammation induced by UV-B exposure might suppress the formation of these ω-hydroxy ceramides in the epidermis.

An interesting finding in our study was that dietary SM significantly suppressed an increase in the mRNA levels of inflammation-associated genes. One potential explanation of this result is that sphingolipids may themselves be a source of anti-inflammatory properties, as would be suggested by the potent protein kinase C inhibitory activity of sphingosine *in vitro* [32]. Furthermore, treatment with sphingosine inhibited phorbol ester-induced skin inflammation via inactivation of protein kinase C in mice [33]. Dietary glucosylceramide also suppressed the release of IL-1α in the skin of mice bred under dry skin conditions [34]. Thus, dietary SM might attenuate skin inflammation and the decrease in covalently-bound ω-hydroxy ceramides, which together would result in improved skin barrier function.

Another possible explanation of the changes in inflammation-related gene expression is that dietary SM may facilitate the formation of the CE. The epidermal CE is a complex protein-lipid composite that replaces the plasma membrane of terminally differentiated keratinocytes [35]. This lamellar structure is crucial for the barrier function of skin and can prevent the loss of water and provide protection from environmental hazards. Loricrin is the major component of the CE in the epidermis, contributing as much as 70% of the CE mass [36–38], and provides a scaffold for the CE. Loricrin-deficient mice, furthermore, showed delayed formation of the skin barrier during embryonic development [39]. Previous reports showed that a single exposure to UV-B down-regulated loricrin, keratin 10, and filaggrin expression *in vitro* [40], which is in agreement with our *in vivo* results that also demonstrated that UV-B irradiation decreased loricrin mRNA expression levels.

Dietary SM suppressed these decreases in the mRNA level of loricrin following UV-B exposure, while an increase was seen in the mRNA levels of TGase-3. TGases are expressed mainly in the skin epidermis and play roles in CE formation during keratinocyte differentiation [21, 41]. TGase-3 in particular promotes the cross-linking of loricrin and small proline-rich proteins to form small interchain oligomers that are then permanently cross-linked to the developing CE [42]. Previous research reported that sphingosine, which is the major metabolite of sphingolipids, enhanced CE production and TGase-3 expression *in vitro* [43]. Therefore, the sphingoid base might contribute to an increase in CE formation due to an activation of TGase-3. Consequently, we showed here that dietary SM stimulated mRNA levels of both loricrin and TGase-3, and enhanced the production of the CE in UV-B damaged-skin, which in turn would improve skin barrier function. On the other hand, milk derived-phospholipid contains glycerophospholipids besides sphingolipids such as SM. Glycerophospholipids also have a potential to effect for the skin. Furthermore, previous reports revealed that oral supplementation with glucosylceramide improved skin barrier function [21, 43]. Hydrolyzed SM form, such as ceramide, may represent potential active agent because SM and glucosylceramide are absorbed as the hydrolysate through intestines [44, 45]. Further studies are needed to clarify contribution to the skin by type of sphingolipids.

Taken together, dietary SM prevented disruption of skin barrier function of hairless mouse induced by UV-B irradiation. Furthermore, the administration of SM increased the strength of epidermal structures, due to an increase in both covalently-bound ceramide and CE formation. Further human studies needed because human has more complex SC and SC lipid composition with more comprehensive biosynthesis pathways. Dietary SM might thus modulate epidermal structures and prevent skin photodamage induced by UV-B exposure.
